# Hepatotoxicity, Nephrotoxicity and Oxidative Stress in Rat Testis Following Exposure to Haloxyfop-*p*-methyl Ester, an Aryloxyphenoxypropionate Herbicide

**DOI:** 10.3390/toxics3040373

**Published:** 2015-10-15

**Authors:** Ebenezer Tunde Olayinka, Ayokanmi Ore

**Affiliations:** Biochemistry Unit, Department of Chemical Sciences, Ajayi Crowther University, PMB 1066, Oyo, Oyo State 211213, Nigeria; E-Mail: oreayokanmi@gmail.com

**Keywords:** aryloxyphenoxypropionate, haloxyfop-*p*-methyl ester, hepatotoxicity, nephrotoxicity, oxidative stress, testicular function, rat

## Abstract

Haloxyfop-*p*-methyl ester (HPME) ((*R*)-2-{4-[3-chloro-5-(trifluoromethyl)-2-pyridyloxy]phenoxy}propionic acid), is a selective aryloxyphenoxypropionate (AOPP) herbicide. It exerts phytotoxicity through inhibition of lipid metabolism and induction of oxidative stress in susceptible plants. This study investigated the toxicological potentials of HPME in rats. Twenty-four male Wistar rats (170–210 g) were randomized into four groups (I–IV). Group I (control) received 1 mL of distilled water, while animals in Groups II, III and IV received 6.75, 13.5 and 27 mg/kg body weight HPME, respectively, for 21 days. There was a significant (*p* < 0.05) increase in renal and hepatic function biomarkers (urea, creatinine, total bilirubin, ALP, ALT, AST) in the plasma of treated animals compared to control. Levels of testicular antioxidants, ascorbic acid and glutathione, and activities of glutathione-*S*-transferase, superoxide dismutase and catalase were reduced significantly after 21 days of HPME administration in a dose-dependent manner. The testicular malondialdehyde level increased significantly in the HPME-treated rats relative to the control. A significant decrease in testicular lactate dehydrogenase, acid phosphatase and γ-glutamyl transferase was also observed in HPME-treated animals. Testicular histology revealed severe interstitial edema and sections of seminiferous tubules with necrotic and eroded germinal epithelium in the HPME-treated rats. Overall, data from this study suggest that HPME altered hepatic and renal function and induced oxidative stress and morphological changes in the testis of rats.

## 1. Introduction

In the course of the last decade, applications of pesticides have increased steadily in Nigeria and in most developing countries as part of the effort to improve food production. Due to intensive applications, large amounts of these substances are released into the environment and find their way to non-target organisms, resulting in toxic effects [1]. Moreover, occupational exposure to these compounds may occur in agricultural workers, workers in the pesticide industry and retailers of the products from improper handling [2]. Some of the commonly-reported adverse effects of human and animal exposures to herbicides include oxidative stress, immunomodulation, disruption of reproductive functions [3] and histopathological alterations in vital organs [3].

**Figure 1:**
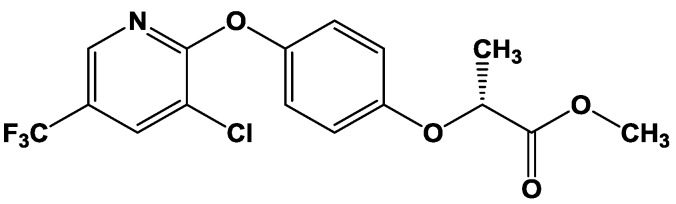
Haloxyfop-*p*-methyl ester methyl ((*R*)-2-{4-[3-chloro-5-(trifluoromethyl)-2-pyridyloxy]phenoxy}propionate).

Most of the widely-applied herbicides in Nigeria include the derivatives of aryloxyphenoxypropionates (AOPPs) [4], including haloxyfop-*p*-methyl ester (Figure 1). Haloxyfop-*p-*methyl ester (HPME) is employed for the control of broadleaf and grassy weeds in corn, sorghum, sugarcane, pineapple, Christmas trees and other crops. Although limited information exists on the metabolism of HPME in both plants and animals, haloxyfop-*p* has been recovered as the major metabolite in plants and in soil [5]. The phytotoxicity mechanism of HPME involves inhibition of acetyl CoA carboxylase (an enzyme of lipid biosynthetic pathway) and induction of oxidative stress in susceptible plants species [6].

Although the mechanism of toxicity of HPME in animals is yet to be fully clarified, most of the reported toxicities of herbicides in mammals involved the generation of reactive oxygen species (ROS) or the disruption of redox balance in tissues [7]. Previously-studied AOPPs are known to generate free radicals and reactive oxygen species, leading to oxidative stress in susceptible plants and in non-target animal species [8,9]. There is increasing evidence suggesting that environmental contaminants play important roles in the modulation of tissue redox homeostasis [10]. Free radicals and reactive oxygen species (ROS) generated by these toxicants are highly reactive, and they can predispose the tissues to lipid peroxidation and tissue damages [11]. To maintain the physiological redox balance, enzymatic and non-enzymatic antioxidants are essential in defending tissues against the deleterious effects of ROS [12]. Generally, animal tissues possess the activities of enzymic antioxidants, like catalase (CAT), superoxide dismutase (SOD), glutathione peroxidase (GPx), *etc.*, and non-enzymic antioxidants, like ascorbic acid (AA) and reduced glutathione (GSH). In addition, these antioxidants are also valuable indicators of oxidative damage and toxicities from environmental toxicants [13].

Currently, little or no information exists on the acute and sub-acute toxicity of haloxyfop-*p*-methyl ester in non-target organisms, such as mammals. Considering the intensive application of this herbicide for domestic and agricultural purposes, an evaluation of its toxicological effects is of great importance for public health. Therefore, it was considered worthwhile to investigate the potential influence of haloxyfop-*p*-methyl ester on testicular function endpoints and the antioxidant defense mechanism in the testis of rats. In addition, renal and hepatic function parameters were also investigated following oral exposure to three different doses of the herbicide.

## 2. Materials and Methods

### 2.1. Chemicals and Reagents

Haloxyfop-*p*-methyl ester (Gallant Super^®^) was purchased from Zhejiang Jinfanda Biochemical Co., Ltd., Zhejiang, China. Glutathione, 1-chloro-2,4-dinitrobenzene(CDNB), 5,5′-dithio-bis-2-nitrobenzoic acid (DTNB), epinephrine and hydrogen peroxide (H_2_O_2_) were obtained from Sigma^®^ Chemical Company, London, U.K. Assay kits for alanine aminotransferase (ALT), aspartate aminotransferase (AST), alkaline phosphatase (ALP), acid phosphatase (ACP), gamma glutamyl transferase (γ-GT), urea, creatinine and bilirubin were products of RANDOX^®^ Laboratories Ltd., Antrim, U.K. The assay kit for lactate dehydrogenase (LDH) was obtained from Cypress Diagnostics, Langdorp, Belgium. All other reagents employed were of analytical grade and highest purity.

### 2.2. Animal Selection and Care

Twenty-four male Wistar rats (170–210 g) were used in this study. They were acquired from the animal breeding unit, Department of Chemical Sciences, Ajayi Crowther University, Oyo, Nigeria. The animals were acclimatized to laboratory conditions for two weeks preceding the start of the study. The rats were contained in wire-meshed cages and provided with food and water *ad libitum*. They were kept at normal conditions of temperature and humidity and fed with commercial rat diet (Ladokun^®^ Feeds, Nigeria Ltd., Ibadan, Nigeria). Handling of the experimental animals is consistent with international principles on the care and use of experimental animals [14].

### 2.3. Experimental Design

The animals were randomized into four experimental groups (I–IV) of six animals each. Animals in each group were treated as presented in Table 1. The respective doses were delivered in 1 mL solution, once daily by oral intubation, for a period of 21 days.

**Table 1:** Experimental design.

Treatment groups	Treatments
I	Control
II	6.75 mg/kg bw HPME
III	13.5 mg/kg bw HPME
IV	27.0 mg/kg bw HPME

### 2.4. Collection of Blood and Testis

Blood samples were obtained from each rat 24 h after the last treatment, through retro orbitals plexus. Blood samples were collected in heparinized (Li heparin) sample tubes. Animals were thereafter euthanized, and testes were carefully removed from each animal for preparation of the testicular homogenates and histopathological analysis. Testicular samples meant for histopathological analysis were immediately fixed in Bouin’s solution for 24 h.

### 2.5. Preparation of Plasma and Sub-Cellular Fractions of Testicular Homogenates

Blood samples were centrifuged at 4000 rpm for 5 min in a bench centrifuge (Analytika, Athens, Greece). Plasma obtained (the supernatant) was stored at −4 °C for subsequent biochemical assays. Testis was rinsed in ice-cold 1.15% KCl and homogenized in 4 volumes of ice-cold 0.01 M potassium phosphate buffer (pH 7.4). The homogenate was centrifuged at 12,500× *g* for 15 min at −4 °C (Eppendorf, Stevenage, GB, U.K.), and the post-mitochondrial fraction (PMF) was used for subsequent biochemical assays.

### 2.6. Assay of the Biomarkers of Hepatotoxicity

Plasma total bilirubin and activities of alanine aminotransferase (ALT), alkaline phosphatase (ALP) and aspartate aminotransferase (AST) were assayed using RANDOX^®^ assay kits following the manufacturer’s procedure. The procedure of Tietz [15] was followed in the determination of plasma TBILI level. The dimethyl sulfoxide formed is a colored compound absorbing maximally at 550 nm. The activity of ALP was assayed following the principles of Tietz *et al.* [16] based on the hydrolysis of *p*-nitrophenyl phosphate to *p*-nitrophenol. The *p*-nitrophenol formed is yellow in color, and its intensity was observed at 405 nm to give a measure of ALP activity. Plasma ALT and AST activities were assayed by the method of Reltman and Frankel [17]. ALT activity was determined by observing the concentration of pyruvate hydrazone generated with 2,4-dinitrophenylhydrazine at a wavelength of 546 nm. Plasma AST activity was determined by monitoring the concentration of oxaloacetate hydrazone produced with 2,4-dinitrophenylhydrazine at a wavelength of 546 nm.

### 2.7. Assay of the Biomarkers of Nephrotoxicity

The levels of urea and creatinine in the plasma were assayed using RANDOX^®^ assay kits following the manufacturer’s protocol. The method for creatinine assays was done according to the colorimetric alkaline picrate method [15]. The creatinine-picrate complex formed has maximum absorbance at 492 nm. Plasma urea concentration was assayed by the procedure of Jaffe [18], with a diazine chromogen generated absorbing maximally at a wavelength of 540 nm.

### 2.8. Assay of the Testicular Protein Content

The protein content of the testicular PMF was assayed by the biuret procedure of Gornall *et al.* [19] using bovine serum albumin (BSA) as a standard protein.

### 2.9. Assay of the Biomarkers of Testicular Function

Gamma glutamyl transferase (γ-GT), acid phosphatase (ACP) and lactate dehydrogenase (LDH) were assayed in the testicular PMF using RANDOX^®^ diagnostic kits according to the manufacturer’s procedure. Testicular γ-GT activity was determined as described by Szasz [20]. ACP activity was determined by the method of Tietz [15] using *p*-nitrophenyl phosphate as the substrate. LDH activity was determined based on the method of Cabaud and Wroblewski [21].

### 2.10. Assay of the Testicular Non-Enzymatic Antioxidants

#### 2.10.1. Assay of the Testicular Glutathione Level

The level of GSH in the testicular PMF was determined spectrophotometrically according to Jollow *et al.* [22]. The colored product (2-nitro-5-thiobenzoic acid) produced from the reaction of Ellman’s reagent (5,5′-dithiobis-(2-nitrobenzoic acid), DTNB) with GSH has a molar absorption at 412 nm.

#### 2.10.2. Assay of the Testicular Ascorbic Acid Level

The ascorbic acid (AA) content was determined according to Jagota and Dani [23]. AA in biological samples reacts with Folin-Ciocalteu (Folin-phenol) reagent to give a blue color, which has maximum absorbance at 760 nm.

### 2.11. Assay of the Testicular Enzymic Antioxidants

#### 2.11.1. Assay of the Testicular Glutathione-*S*-Transferase Activity

The activity of GST in the testicular PMF was determined by the principle of Habig *et al.* [24] using 1-chloro-2,4-dinitrobenzene (CDNB) as the substrate.

#### 2.11.2. Assay of the Testicular Superoxide Dismutase Activity

The activity of SOD in the testicular PMF was determined by the procedure of Misra and Fridovich [25]. This was achieved by monitoring the inhibition of auto-oxidation of epinephrine at alkaline pH (pH 10.2).

#### 2.11.3. Assay of the Testicular Catalase Activity

The activity of CAT in the testicular PMF was assayed by the method described by Singha [26]. Dichromate in acetic acid is reduced to chromic acetate when heated in the presence of H_2_O_2_. The chromic acetate formed was measured spectrophotometrically at the wavelength of 570 nm. The activity of CAT in the sample was expressed as micromoles of H_2_O_2_ consumed per min per mg protein.

### 2.12. Assay of the Level of Lipid Peroxidation in the Testicular PMF

The level of lipid peroxidation (LPO) in the testis was assayed by the method of Vashney and Kale [27]. Malondialdehyde (MDA; a product of lipid peroxidation) reacts with thiobarbituric acid to yield a pink chromogen, which absorbed maximally at 532 nm.

### 2.13. Testicular Histology

The method described by Baker and Silverton [28] was employed in processing testicular samples for histopathological examinations. Bouin-fixed testicular tissues were dehydrated stepwise in graded ethanol and embedded in paraffin wax. A thin section (5-μm thickness) was made from the mid-portion of each sample and stained with hematoxylin and eosin, followed by examination under a light microscope.

### 2.14. Statistical Analysis

Data are presented as the mean ± standard deviation (SD) of six replicates. Statistical significance was determined by one-way analysis of variance (ANOVA) and complemented with Duncan’s multiple comparison between control and treated animals in all groups using SigmaPlot^®^ statistical software (Systat^®^ Software Inc., San Jose, CA, USA). *p*-values less than 0.05 (*p* < 0.05) were considered statistically significant.

## 3. Results

### 3.1. Influence of Haloxyfop-p-methyl Ester on Hepatic Function Markers in the Plasma of Rat

Data presented in Table 2 represent the hepatic function parameters of control and HPME-treated animals. Plasma bilirubin increased significantly (*p* < 0.05) in animals treated with HPME in a dose-dependent manner by 28%, 62% and 97%, respectively. The activities of the marker enzymes ALP, ALT and AST also increased significantly in the plasma of HPME-treated animals compared to the control. The activity of ALP increased by 35%, 46% and 67%, respectively, in the groups given 6.75, 13.5 and 27 mg/kg bw HPME, respectively. The plasma activity of ALT also increased significantly by 18%, 35% and 49%, respectively, in the HPME-treated animals compared to the control. In a similar manner, HPME caused a significant increase in plasma AST activity by 8%, 20% and 26%, respectively, relative to the control.

**Table 2:** Effect of haloxyfop-*p* methyl ester on plasma biomarkers of hepatic function in rat.

Treatment groups	Bilirubin (mg/dL)	ALP (U/L)	ALT (U/L)	AST (U/L)
0 (Control)	0.29 ± 0.01	262.8 ± 8.6	30.4 ± 3.1	61.2 ± 5.6
6.75 mg/kg bw HPME	0.37 ± 0.01 (28%) *	355.4 ± 9.1 (35%) *	36.0 ± 2.6 (18%) *	66.4 ± 4.1 (8%) *
13.50 mg/kg bw HPME	0.47 ± 0.02 (62%) *	384.2 ± 6.6 (46%) *	41.1 ± 2.3 (35%) *	73.4 ± 5.1 (20%) *
27.00 mg/kg bw HPME	0.57 ± 0.01 (97%) *	438.8 ± 7.4 (67%) *	45.2 ± 3.6 (49%) *	77.0 ± 2.7 (26%) *

Values are the means ± SD for six replicates; * values significantly different from the control at *p* ˂ 0.05; values in parenthesis represent the percentage (%) increase compared to the control.

### 3.2. Influence of Haloxyfop-p-methyl Ester on Renal Function Markers in the Plasma of Rat

Data presented in Table 3 show the effect of different doses of HPME on the plasma level of urea and creatinine in rat. Plasma urea level increased significantly (*p* < 0.05) in the HPME-treated animals by 33%, 70% and 96%, respectively, when compared to the control. Plasma creatinine also increased in a similar way by 33%, 57% and 73%, respectively, in the treated groups relative to the control.

**Table 3:** Effect of haloxyfop-*p* methyl ester on biomarkers of the renal function in rat.

Treatment groups	Urea (mg/ dL)	Creatinine (mg/ dL)
0 (Control)	18.0 ± 2.3	0.42 ± 0.06
6.75 mg/kg bw HPME	24.1 ± 1.8 (33%) *	0.56 ± 0.03 (33%) *
13.50 mg/kg bw HPME	30.6 ± 2.4 (70%) *	0.66 ± 0.03 (57%) *
27.00 mg/kg bw HPME	35.3 ± 3.1 (96%) *	0.73 ± 0.07 (73%) *

Values are the means ± SD for six replicates; * values significantly different from the control at *p* ˂ 0.05; values in parenthesis represent the percentage (%) increase compared to the control.

### 3.3. Influence of Haloxyfop-p-methyl Ester on Biomarkers of Testicular Function in Rat

**Figure 2:**
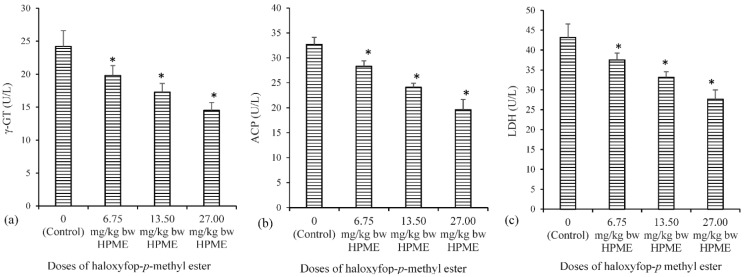
The influence of haloxyfop-*p*-methyl ester on biomarkers of testicular function in rat: (**a**) γ-glutamyl transferase (γ-GT); (**b**) acid phosphatase (ACP); and (**c**) lactate dehydrogenase (LDH); data represent the mean of six replicates ± standard deviation. * Significance of difference at *p* < 0.05 (Duncan’s multiple comparison of control and HPME-treated rats).

The effect of HPME on testicular activities of acid phosphatase (ACP), lactate dehydrogenase (LDH) and γ-glutamyl transferase (γ-GT) are presented in Figure 2. Testicular γ-GT, ACP and LDH activities showed a significant (*p* < 0.05) decrease in all of the HPME-treated groups compared to the corresponding control values.

### 3.4. Effect of Haloxyfop-p-methyl Ester on Biomarkers of Oxidative Stress in the Testis of Rat

**Figure 3:**
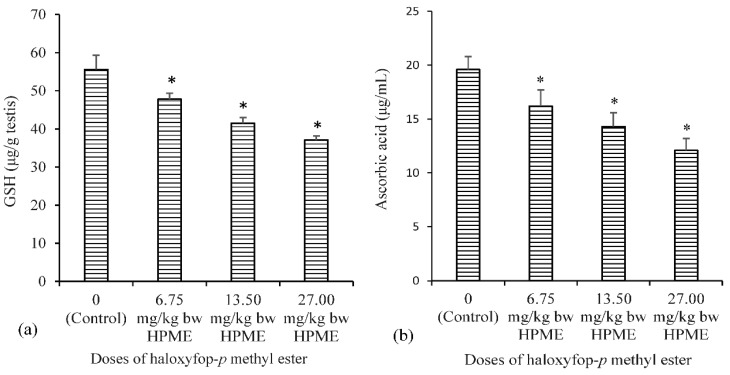
The influence of haloxyfop-*p*-methyl ester on the levels of non-enzymatic antioxidants in rat testes: (**a**) reduced glutathione; and (**b**) ascorbic acid (AA); data represent the mean of six replicates ± standard deviation. * Significance of difference at *p* < 0.05 (Duncan’s multiple comparison of control and HPME-treated rats).

**Figure 4:**
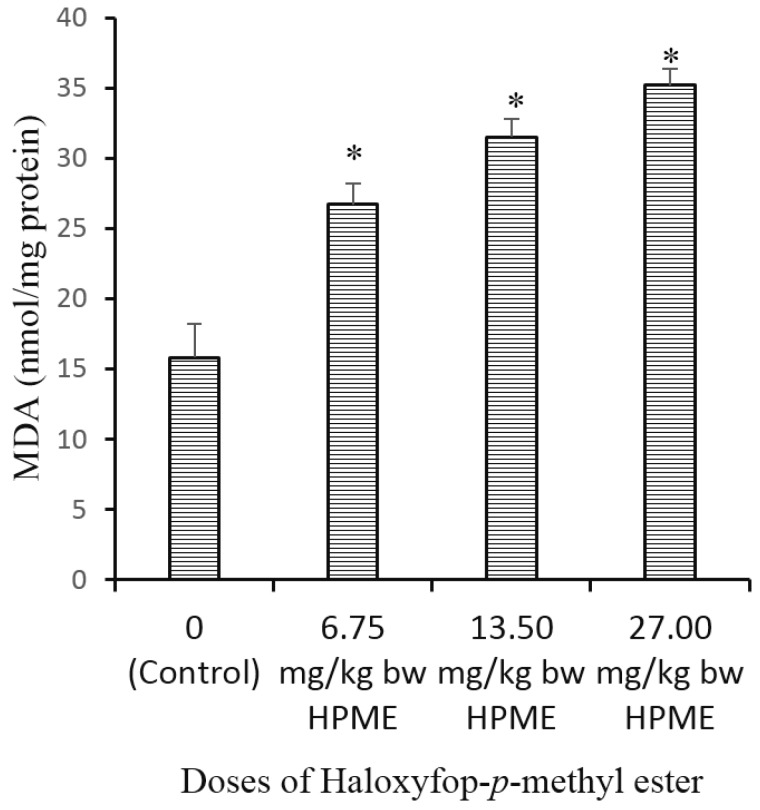
The influence of haloxyfop-*p*-methyl ester on the levels of malondialdehyde (MDA) in rat testis; data represent the mean of six replicates ± standard deviation. * Significance of difference at *p* < 0.05 (Duncan’s multiple comparison of control and HPME-treated rats).

The effect of different doses of HPME on testicular biomarkers of oxidative stress is presented in Figure 3 (non-enzymic antioxidants), Figure 4 (level of lipid peroxidation) and Table 4 (enzymic antioxidants). A significant reduction in the testicular antioxidant status was noted, following exposure to HPME. The testicular levels of glutathione and ascorbic acid (the non-enzymic antioxidants) were significantly (*p* < 0.05) decreased in HPME-exposed rats in a dose-dependent manner compared to controls (Figure 3a,b). Testicular activities of the enzymic antioxidants GST, SOD and CAT were also significantly decreased in rats administered various doses of HPME (Table 4). GST activity decreased by 19%, 30% and 46% in the groups administered different doses of HPME. The activity of testicular SOD also decrease by 23%, 37% and 49% in the HPME-treated groups. Testicular CAT activity was significantly reduced by 24%, 44% and 60%, respectively. HPME also caused a significant increase in the level of testicular malondialdehyde (MDA) level in the HPME-treated animals in a dose-dependent manner relative to the control (Figure 4).

**Table 4:** Influence of haloxyfop-*p*-methyl ester on enzymatic antioxidants in the testis of rat.

Treatment groups	GST (nM/min/mg protein)	SOD (units/mg protein)	CAT (μM H_2_O_2_ consumed/min/mg protein)
0 (Control)	22.6 ± 1.7	8.6 ± 0.7	13.9 ± 1.2
6.75 mg/kg bw HPME	18.2 ± 1.8 (19%) *	6.6 ± 0.5 (23%) *	10.6 ± 0.7 (24%) *
13.50 mg/kg bw HPME	15.8 ± 2.1 (30%) *	5.4 ± 0.6 (37%) *	8.2 ± 0.5 (44%) *
27.00 mg/kg bw HPME	12.1 ± 1.5 (46%) *	4.4 ± 0.4 (49%) *	6.1 ± 0.5 (60%) *

Values are the means ± SD for six replicates; * values significantly different from the control at *p* ˂ 0.05; values in parenthesis represent the percentage (%) decrease compared to the control.

### 3.5. Effect of Haloxyfop-p-methyl Ester on Histological Characteristics of Rat Testis

**Figure 5:**
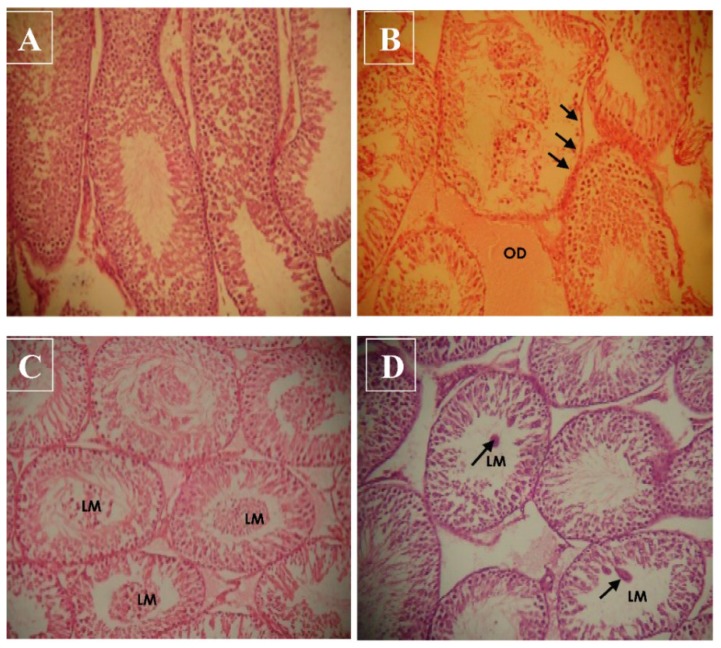
Representative photomicrograph of the testicular sections showing the morphological changes induced by haloxyfop-*p*-methyl ester in the testes of rats (hematoxylin and eosin stain, 200×); control group (**A**) and treated groups at the doses of 6.75 mg/kg bw (**B**), 13.50 mg/kg bw (**C**) and 27.00 mg/kg bw (**D**). (**A**) shows normal testicular structure; in (**B**), there are severe interstitial edemas (OD) and some sections of the seminiferous tubule have necrotic and eroded germinal epithelium; in (**C**), most sections of the seminiferous tubules have immature cells in the lumen (LM); and in (**D**), there are few cellular clumps in the lumen of some of the seminiferous tubules.

The representative photomicrographs of the testicular sections from rats in the control and HPME-treated groups are presented in Figure 5. Histopathological examination of testicular sections from rats in the control group demonstrated normal and well-defined cellular arrangements. However, exposure to 18.75 mg/kg bw HPME caused severe interstitial edema (OD) (Figure 5b). In addition, some sections of the seminiferous tubule displayed a necrotic and eroded germinal epithelium. In Figure 5c (37.50 mg/kg bw HPME), most sections of the seminiferous tubules have immature cells in the lumen (LM), and in Figure 5d (75 mg/kg bw HPME), there are few cellular clumps in the lumen of some of the seminiferous tubules.

## 4. Discussion

The domestic, industrial and agricultural applications of herbicides have increased in the last decade, leading to better weed control and high economic gains in agriculture. Herbicides are developed, produced, packed and transported under strictly-regulated processes in order to minimize their impact on the environment and human health. Nevertheless, serious concerns have been raised about their potential public health risks from environmental contamination and occupational exposure [29]. Occupational exposure to herbicides may include the exposure of agricultural workers in farms and fields and workers in the pesticide industry, as well as retailers of the products [30]. The general population can also be exposed to residues of herbicides through food, contaminated water and domestic use. Exposure to these substances can result in a number of health effects depending on the toxicological properties of the substance involved, the concentration of the herbicide taken, the quantity applied in the environment, the half-life and the persistence of the active metabolites in the environment. Therefore, the assessment of the environmental and health effects of herbicides are very crucial for public health.

Most of the reported adverse effects of herbicides include organ toxicity, oxidative damage to tissues, endocrine disruptions and reproductive toxicities in human and animal studies [31]. The present study evaluates the potential effects of haloxyfop-*p*-methyl ester (HPME) on renal, hepatic and testicular functions in rat. The effects of the herbicide on biomarkers of oxidative stress were also considered in the testis of rat. Following oral exposure, toxicants are generally distributed and metabolized in the liver, which can predispose this organ to chemical-induced toxicity. The plasma level of total bilirubin and activities of the liver marker enzymes ALP, ALT and AST are well-established indices of hepatotoxicity [32]. An increase in the plasma level of total bilirubin (TBILI) and activity of ALP is associated with hepatobiliary damage and hepatic cholestasis [33]. Bilirubin is present in liver, bile, intestines and the reticuloendothelial cells of the spleen, while ALP is associated with the liver cell membrane [34]. The plasma TBILI level and activity of ALP are known to increase in conditions accompanying hepatobiliary damage and leakage of ALP from hepatocytes [35].

The presence of high plasma activities of ALT and AST is an established indicator of hepatocellular damage in human and animal studies [36]. Elevations of ALT and AST activities in the plasma has been linked to hepatocellular damages [37]. In this study, we observed a significant increase in plasma ALT and AST activities, suggesting their leakage from injured hepatocytes [38]. Previous studies have reported a similar increase in the plasma of herbicide-treated rat [39]. The present observation on HPME-induced increase in plasma TBILI and activities of liver markers (ALP, ALT and AST) is in agreement with previous work in our laboratory on the aryloxyphenoxypropionate herbicide fluazifop-*p*-butyl [9].

Plasma levels of urea and creatinine are important biomarkers of renal function in human and animal studies [40]. Data from this study indicate that HPME caused a significant increase in plasma urea and creatinine. An increase in the levels of these substances in the plasma is an indication of the loss of renal function [41]. Besides, earlier studies on phenoxypropionate herbicides observed a similar increase in plasma urea and creatinine [9].

The activities of γ-GT, ACP and LDH in testis are useful indices of testicular function and used in assessing testicular response to toxicants [42]. Testicular γ-GT activity is a useful indicator of Sertoli cell function [43]. γ-GT is involved in the metabolism of GSH, a process that delivers precursor amino acids for intracellular GSH synthesis [44]. A similar decrease in testicular γ-GT activity was also observed in an earlier study on fluazifop-*p* butyl [9]. The activity of ACP is present in lysosomes of Leydig cells and is involved in the removal of unneeded sperm cells [45]. Moreover, the activity of testicular ACP may be used as an indicator of functional spermatogenesis [46]. Reduction in the activity of ACP in the testes of HPME-treated rats is an indication of testicular degeneration and a suppressed lysosomal function [47]. The activity of LDH in testis is associated with the maturation of the germinal epithelial layer of seminiferous tubule. It also plays a role in providing lactate to developing germ cells. LDH activity is also present in the Sertoli cells, where it participates in testicular energy metabolism [45]. The observed decrease in testicular LDH activity is an indication of the interference of HPME with testicular energy metabolism [48].

Industrial and environmental toxicants have been shown to possess the capacity to disrupt male fertility by inducing oxidative stress in the testes [49]. Normal testicular function is dependent on functional redox homeostasis regulated by the presence of enzymes, like SOD, CAT, GST, GPx, *etc.*, and non-enzymic antioxidants, like AA, GSH, *etc.* [50]. The activities of these antioxidants are essential for redox balance and protection of spermatogenic cells, including the maintenance of overall male fertility [51]. The activity of SOD is vital to testicular defense strategy. The testes contain cytosolic (Cu/Zn-SOD) and the mitochondrial (Fe/Mn-SOD), including the unusual extracellular SOD, (SOD-Ex), which is synthesized in the Sertoli and germ cells [52]. The activity of the testicular SOD system is required for the conversion of superoxide radical to hydrogen peroxide (H_2_O_2_) and molecular oxygen [52]. The H_2_O_2_ generated in this process and other biochemical processes is transformed into water and oxygen through the action of CAT [53]. GST is a complex family of proteins that catalyze the conjugation of reduced glutathione to a wide variety of substrates in preparation for elimination from the cell [54]. GST activity is critical in the detoxification of peroxidized lipids, as well as the metabolism of toxicants [54]. However, GST also constitutes a vital component of the antioxidant system in the testis, and it has been reported to be essential for male fertility [55]. The observed pattern of HPME-induced depletion of the testicular enzymic antioxidant system is similar to that reported by Abarikwu *et al.* [56] on atrazine.

The non-enzymic antioxidant molecules, GSH and AA, play important roles in cellular redox balance. They function as free radical scavengers in cells and as the first line of antioxidant defense in tissues. GSH is a cofactor for glutathione peroxidase (GPx) and glutathione-*S*-transferase, and it also participates in free radical scavenging activities in the testis [57]. The decreased levels of GSH after exposure to HPME may be due to the consumption of GSH in the conjugation reaction or a decrease in its biosynthesis and turnover. AA functions in the aqueous environment and is involved in the preservation of tocopherol in cell membranes [53]. The decrease in levels of testicular GSH and AA observed in this study may be due to the presence of free radicals generated by HPME, thus predisposing the testis to oxidative stress [57].

The tissue level of MDA, an indicator of LPO, is a frequently-used marker of oxidative stress and tissue damage *in vivo* [50]. Despite the low oxygen tensions in the testicular micro-environment, this tissue remains vulnerable to oxidative stress due to the presence of unsaturated fatty acids [58]. LPO is a physiological event in normal cells and a well-known mechanism of cellular injury or stress response in animal tissues [59]. In previous studies, an increase in LPO has been observed as one of the testicular toxicity mechanisms induced by phenoxyacetic acid herbicides [60]. The fluidity of biological membranes is owed to the presence of polyunsaturated fatty acids (PUFAs) [61]. Since the testis is rich in PUFAs, it is easily prone to lipid peroxidation [50]. Peroxidation of membrane lipids can result in disruption of cell structural integrity [62] and cell damage. Hence, the increase in MDA levels caused by HPME in the rat testis suggests peroxidation of PUFAs in testicular cells, which can cause impairment of normal testicular and sperm function [63], which is common with phenoxyacetic acid herbicides [64].

In this study, HPME was found to induce various morphological degenerations in rat testis. The changes, which include severe interstitial edema (OD), necrotic and eroded germinal epithelium, the presence of immature germ cells and cellular clumps in the lumen of some of the seminiferous tubules, are similar to those obtained from studies on related AOPP (fluazifop-*p*-butyl) and other phenoxyacetic acid herbicides [9,60].

## 5. Conclusions

Data from this study demonstrated that haloxyfop-*p*-methyl ester (at the doses tested) is capable of inducing testicular oxidative stress and causing impairment of renal and hepatic functions in rat. Oxidative stress plays a role in male fertility, and therefore, appropriate measures should be taken to minimize occupational exposure by applicators. Further studies are required to determine the toxicity of HPME in other non-target animal models, as well as in other tissues.

## Abbreviations

AOPPs	aryloxyphenoxypropionates
HPME	haloxyfop-*p*-methyl ester
CDNB	1-chloro-2,4-dinitrobenzene
DTNB	5,5′-dithio-bis-2-nitrobenzoic acid
bw	body weight
PMF	post mitochondrial fraction
BSA	bovine serum albumin
ddH_2_O	double-distilled water
TBILI	total bilirubin
ALP	alkaline phosphatase
ACP	acid phosphatase
ALT	alanine aminotransferase
AST	aspartate aminotransferase
γ-GT	gamma glutamyl transferase
LDH	lactate dehydrogenase
SOD	superoxide dismutase
CAT	catalase
GPx	glutathione peroxidase
GST	glutathione-*S*-transferase
AA	ascorbic acid
GSH	reduced glutathione
LPO	lipid peroxidation
MDA	malondialdehyde
ROS	reactive oxygen species
H_2_O_2_	hydrogen peroxide
PUFA	polyunsaturated fatty acids
LM	lumen
OD	edema
